# Epidemiological characteristics of carbapenem-resistant *Enterobacteriaceae* collected from 17 hospitals in Nanjing district of China

**DOI:** 10.1186/s13756-019-0674-4

**Published:** 2020-01-13

**Authors:** Hui Zhou, Kui Zhang, Wei Chen, Junhao Chen, Jie Zheng, Chang Liu, Li Cheng, Wanqing Zhou, Han Shen, Xiaoli Cao

**Affiliations:** 1 Department of Laboratory Medicine, Nanjing Drum Tower Hospital, the affiliated Hospital of Nanjing University Medical School, Zhongshan Road 321, Gulou District, Nanjing, Jiangsu People’s Republic of China; 2 Clinical Research Center, the second hospital of Nanjing, Nanjing University of Chinese Medicine, Nanjing, 210003 China

**Keywords:** CRE, Carbapenemase, Plasmid replicon, Sequence type, *Klebsiella pneumoniae*

## Abstract

**Objective:**

In total, 97 carbapenem-resistant *Enterobacteriaceae* (CRE) were collected from 17 hospitals located in Nanjing, Southeast China, and analyzed for epidemiological characteristics.

**Methods:**

Antimicrobial susceptibility was determined; followed by determination of the prevalence of resistance determinants, including extended-spectrum β-lactamase (ESBLs), plasmid-mediated AmpC enzyme (pAmpCs), plasmid-mediated quinolone resistance genes (PMQRs), fosfomycin resistance gene and exogenously acquired 16S rRNA methyltransferase (16S-RMTase) using PCR and DNA sequencing. The sequence types (STs) of CRE were determined by multi-locus sequence typing (MLST). The plasmid profiles were detected by PCR-based replicon typing (PBRT).

**Results:**

All the CRE strains displayed high MIC_50_ and MIC_90_ for nearly all clinical available antibiotics, except for aztreonam/avibactam, minocycline, ceftazidime/avibactam, tigecycline, and colistin. KPC-2 (79.4%) and NDM (19.6%) were the main carbapenemases, CTX-M (76.3%) and SHV (60.8%) were the predominant ESBLs. In addition, *oqxAB* (70.1%) and *qnr* (63.9%) were the major PMQRs; *rmtB* (47.4%) was the main 16S-RMTase; *fosA* (76.3%) and *fosA3* (37.1%) were the fosfomycin resistance gene. PBRT analysis showed presence of IncR (66.0%) and IncFII (64.9%) replicon types in the majority of the isolates, followed by IncFIB (46.4%) and IncX3 (16.5%). The IncFII and IncR replicon-types were found mainly in *K. pneumoniae* (68.8%), whereas the IncX3 replicons dominated in *E. coli* isolates (100.0%). The three dominating MLST-types ST11, ST15 and ST268 comprised 68.0% of the 77 *K. pneumoniae*. Seven distinct STs were identified among 8 *E. coli.*

**Conclusions:**

The treatment for infections caused by CRE isolates is challenged by the presence of multiple resistance determinants and plasmid replicons. Our results highlighted the expansion of *bla*KPC-2 carrying *K. pneumoniae* ST11, the new emergency of single *bla*NDM-5 carrying *K. oxytoca* ST36*,* as well as *bla*IMP-4 and *bla*NDM-1 co-carrying *E. cloacae* ST418, which alert us on the urgency for antimicrobial resistant surveillance, to prevent dissemination of these highly transmissible and dangerous lineages.

## Introduction

With the wide use of carbapenem antibiotics in clinical therapy, carbapenem-resistant *Enterobacteriaceae* (CRE) has dramatically increased and become a serious public health issue [1]. CRE constitutes a large group of bacteria with different mechanisms for drug resistance [2]. Among them, carbapenem resistant *Klebsiella pneumoniae* (CRKP) accounts for approximately 60%, followed by *Escherichia coli* and *Enterobacter cloacae* [3, 4]*.* Epidemiological studies have shown that production of carbapenemase is the main mechanism for carbapenem resistance [5], with *bla*KPC and *bla*NDM being the most prevalent ones in the CRKP and carbapenem resistant *E. coli* (CREC), respectively [6, 7]*.* Recently, the co-occurrence of multiple resistance determinants, even more than 2 carbapenemases in one strain, has been frequently reported [8, 9], alerting us on the importance of resistance surveillance, since the resistant determinants are mainly plasmid-borne with highly transmissible nature [9, 10]. Additionally, the production of extended-spectrum β-lactamases (ESBLs) and/or AmpC enzymes in combination with decreased permeability by mutations in outer membrane proteins OmpK35 and OmpK36, as well as over-expression of efflux pumps play roles in the carbapenem resistance [11, 12]. It is noteworthy that CRE infections are closely associated with high mortality because of limited antimicrobial use [13]. To date, fosfomycin, minocycline, tigecycline, colistin, ceftazidime/avibactam and aztreonam/avibactam have been recommended for treatment of infections caused by CRE [14]. However, data on susceptibilities of these antibiotics on CRE are still limited.

Although CRE has been frequently reported in China [8, 15], most of the analyzed CRE were collected from the Third-Class A General Hospitals, and the information on CRE isolates collected from specialized hospitals, Children’s hospital and level II hospitals was less available. Furthermore, data on non-*K. pneumoniae* CRE are also less available, owing to the focus of globally disseminated *K. pneumoniae*.

In this study, 97 CRE strains were collected from 17 hospitals, including specialized hospitals, Children’s hospital and level II hospitals. The antimicrobial susceptibility, resistant determinants, sequence types (STs), as well as plasmid replicons, were analyzed to investigate the epidemiological characteristics of these CRE.

## Materials and methods

### Strain collection

Totally, 97 CRE were collected from 17 hospitals in Nanjing district, among them, 77 were *Klebsiella pneumoniae*, 11 *Escherichia coli*, 4 *Citrobacter freundii*, 2 *Escherichia cloacae*, 2 *Klebsiella oxytoca* and 1 *Serratia marcescens*. The following hospitals were involved in this investigation: Nanjing Drum Tower Hospital (*n* = 20), Second Affiliated Hospital of Nanjing Medical University (*n* = 13), Nanjing Maternal and Child Health Hospital (*n* = 2), Jiangning Branch of Zhongda Hospital Affiliated to Southeast University (*n* = 1), Nanjing Meishan Hospital (*n* = 3), Nanjing Liuhe Hospital (*n* = 3), Nanjing First Hospital (*n* = 5), Nanjing jinyu Hospital (*n* = 4), Nanjing Brain Hospital (*n* = 7), Nanjing Lishui Hospital (*n* = 1), Nanjing Dachang Hospital (*n* = 9), Nanjing Thoracic Hospital (*n* = 7), Nanjing Children’s Hospital (*n* = 6), Nanjing Traditional Chinese Medicine Hospital (*n* = 4), Gaochun People’s Hospital (*n* = 2), Nanjing Mingji Hospital l (*n* = 8), and Jiangbei People’s Hospital (*n* = 2). All strains were confirmed by VITEK 2.0 or ATB 32E (bioMérieux. Firenze, Italy). The strains were collected from the following samples: sputum (*n* = 34), urine (*n* = 13), secretion (*n* = 5), blood (*n* = 3), bile (*n* = 1), and pus (*n* = 1), others remained unknown.

### MIC determination

In total, 27 antimicrobial agents were used to test the susceptibilities of the CRE. Among them, 25 were tested by micro-broth dilution method, including ertapenem, imipenem, meropenem, cefepime, ceftazidime, cefotaxime, cefuroxime, cefazolin, cefmetazole, piperacillin/tazobactam, amikacin, gentamicin, funantuoyin, trimethoprim and sulphame-thoxazole, aztreonam, piperacillin, ciprofloxacin, levofloxacin, aztreonam/avibactam, ceftazidime/avibactam, tigecycline, and colistin. The susceptibilities toward fosfomycin and minocycline were determined by Kirby-Bauer method. Results were interpreted according to the guideline of CLSI 2019 [16]. Considering the absence of CLSI breakpoints for interpretation of tigecycline and colistin results, the current European Committee on Antimicrobial Susceptibility Testing (EUCAST) (www.eucast.org) guidelines were used. For tigecycline, a cutoff MIC of ≤1 μg/ml and > 2 μg/ml was used as the susceptibility and resistance breakpoints, respectively. For colistin, a cutoff MIC of ≤2 μg/ml was taken as susceptibility breakpoint. The *E. coli* ATCC25922 was used as quality control. Carbapenem resistance was defined as a MIC of ≥2 μg/ml for ertapenem or a MIC of ≥4 μg/ml for imipenem or meropenem.

### Detection of resistant determinants

DNA templates were prepared by the boiling method. All the 97 strains were detected by PCR for carbapenemase encoding genes (*bla*KPC, *blaA*IM, *bla*SIM, *bla*SPM, *bla*VIM, *bla*IMP, *bla*OXA, *bla*DIM, *bla*NDM, *bla*GIM, and *bla*GES), PMQR *(qnrA, qnrB, qnrC, qnrD, qnrS, aac (6′)-Ib-cr, and qepA*), *bla*ESBL encoding genes (*bla*CTX, *bla*TEM, *bla*SHV, *bla*OXA, *bla*VEB, and *bla*PER), 16S-RMTases encoding genes (*armA, npmA, rmtA, rmtB, rmtC, rmtD*, and *rmtE*) and pAmpC encoding genes (*bla*EBC, *bla*MOX, *bla*ACC, *bla*FOX, *bla*DHA, and *bla*CIT) according to the methods described previously [17–21]. The purified PCR products were sent to the Qingke Biotechnology Co., Ltd. (Nanjing, China) for sequencing. Sequences were analyzed by using the Chromas-Pro application and BLAST (www.ncbi.nlm.nih.gov/BLAST), and the subtypes of β-lactamase genes were confirmed by referring to the Lahey system (www.lahey.org/studies/).

### Multi-locus sequence typing

The STs of CRE were determined by multi-locus sequence typing (MLST). Seven CRKP housekeeping genes, including *gapA*, *infB*, *mdh*, *pgi*, *phoE*, *rpoB*, and *tonB* were amplified and sequenced according to Yang et al. [22]. The MLST database (www.pasteur.fr/mlst/Kpneumoniae.html) was used to assign the alleles and STs. For *E. coli*, the 7 housekeeping genes, including *adk*, *fumC*, *gyrB*, *icd*, *mdh*, *purA*, and *recA* were amplified and analyzed according to the protocol available at https://bigsdb.pasteur.fr/ecoli/ecoli.html. The clonal lineages of *E. cloacae* were determined by analyzing the 7 housekeeping genes (*dnaA*, *fusA*, *gyrB*, *leuS*, *pyrG*, *rplB*, and *rpoB*) following the scheme developed by Miyoshi-Akiyama et al. [23], and the STs were assigned according to the protocols on the MLST website (http://pubmlst.org/ecloacae/). The MLST analysis of *C. freundii* were performed according to the protocol provided on the website (https://pubmlst.org/cfreundii/), with the housekeeping genes *aspC*, *clpX*, *fadD*, *mdh*, *arcA*, *dnaG* and *lysP*. Additionally, the STs of the *K. oxytoca* was analyzed by assigning the definite 7 housekeeping genes (*gapA*, *infB*, *mdh*, *pgi*, *phoE*, *rpoB*, and *tonB*) according to the protocol (https://pubmlst.org/koxytoca/).

### PCR-based replicon typing

In order to determine the distribution of plasmid incompatibility groups among the CRE, thirty different plasmid replicons including HI1, HI2, I1, I2, X1, X2, X3, X4, L, M, N, FIA, FIB, FIC, FII, FIIs, FIIk, FIB-KN, FIB-KQ, W, Y, P1, A/C, T, K, U, R, B/O, HIB-M and FIB-M were determined by using PCR-based replicon typing (PBRT)-KIT 2.0 (DIATHEVA, Italy).

### Statistical analysis

SPSS software (20.0) was used to implement statistical analysis. The differences on distribution of resistant determinants/plasmid replicons between bacteria were analyzed by Chi-square test, and the differences were considered to be significant when *p* value was less than 0.05; The differences on distribution of antimicrobial resistance determinants and plasmid replicons among CRE were analyzed by McNemar test. The distribution rates were considered to be the same when p value was more than 0.05.

## Results

### MIC determination

On the whole, none of the 97 CRE strains were susceptible to β-lactam antibiotics, including imipenem, meropenem, ertapenem, cefepime, ceftazidime, cefotaxime, cefuroxime, cefazolin, piperacillin, ciprofloxacin and levofloxacin. The non-susceptible rates to aztreonam, gentamycin, amikacin were 96.9, 79.4 and 61.9%, respectively. Meanwhile, 86.6% of the CRE were non-susceptible to trimethoprim-sulfamethoxazole and 59.8% to nitrofurantoin. In addition, 31 (32.0%) strains were non-susceptible towards minocycline and 59 (60.8%) to fosfomycin. The good side was that most CRE were still susceptible to aztreonam/avibactam and ceftazidime/avibactam, colistin and tigecycline, with susceptibility rates being 96.9, 78.4, 95.9 and 96.9%, respectively. The relative MIC50 and MIC90 were summarized in Table 1.

**Table 1:** The MIC_50_ and MIC_90_ of the carbapenem resistant *enterobacteraceae* isolates

Antibiotics	MIC_50_	MIC_90_	Range (μg/ml)
ertapenem	> 32	> 32	0.25–32
imipenem	> 16	> 16	0.125–16
meropenem	> 16	> 16	0.125–16
cefepime	> 32	> 32	0.25–32
ceftazidime	> 32	> 32	0.25–32
cefotaxime	> 32	> 32	0.25–32
cefuroxim	> 64	> 64	0.5–64
cefazolin	> 32	> 32	0.25–32
cefmetazole	> 64	> 64	0.5–64
piperacillin/tazobactam	> 256/4	> 256/4	2/4–245/4
amikacin	> 128	> 128	1–128
gentamicin	> 128	> 128	1–128
funantuoyin	> 128	> 128	1–128
trimethoprim and sulphame-thoxazole	2	> 32	0.25/4.75–32/608
aztreonam	> 128	> 128	1–128
piperacillin	> 256	> 256	2–256
ciprofloxacin	> 8	> 8	0.06–8
levofloxacin	> 16	> 16	0.125–8
aztreonam/avibactam	0.5	2	0.25–32
ceftazidime/avibactam	0.25	1	0.25–32
tigecycline	0.5	1	0.125–16
colistin	0.5	0.5	0.125–16

### Prevalence of resistance determinants

The prevalence of resistance determinants was shown in Table 2. Among the 97 CRE strains, 92 (94.8%) CRE carried carbapenemase encoding genes, which were 77 *bla*KPC-2, 19 *bla*NDM and 2 *bla*IMP-4. Ninety-three (95.9%) CRE were found to co-carry more than three resistance determinants. Remarkably, three *E. coli* co-carried *bla*KPC-2 and *bla*NDM-5. Co-existence of *bla*IMP-4 and *bla*KPC-2, and co-occurrence of *bla*KPC-2 and *bla*NDM-5 were also found in the two *K. pneumoniae* isolates. An *E. cloacae* was found to simultaneously carry *bla*NDM-1 and *bla*IMP-4.

**Table 2:** The distribution of resistance determinants among the carbapenem resistance *enterobacteraceae* isolates

Resistant determinants	*K. pneumoniae* (*n* = 77)	*K. oxytoca* (*n* = 2)	*E. coli* (*n* = 11)	*S. marcescens* (*n* = 1)	*C. freundii* (*n* = 4)	*E. cloacae* (*n* = 2)	*Enterobacter* (*n* = 20)	*P* value (*K. pneumoniae* Vs *E. coli*)	*P* value (*K. pneumoniae* Vs *Enterobacter*)
*bla*KPC-2 (*n* = 77)	70	0	3	1	2	1	7	0.000	0.000
*bla*NDM (*n* = 19)	5	1	11	0	1	1	14	0.000	0.000
*bla*CTX-M (*n* = 74)	59	0	10	1	4	0	15	0.493	0.879
*bla*CTX-M-15 (*n* = 18)	16	0	2	0	0	0	2	1.000	0.434
*bla*CTX-M-65 (*n* = 34)	32	0	1	0	1	0	2	0.081	0.018
*bla*SHV (*n* = 59)	49	0	5	0	3	2	10	0.247	0.266
*bla*SHV-11(*n* = 23)	18	0	3	0	1	1	5	1.000	0.879
*bla*TEM (*n* = 73)	60	1	8	0	2	2	13	1.000	0.233
*bla*TEM-1B(*n* = 71)	60	1	7	0	1	2	11	0.508	0.039
*bla*OXA (*n* = 16)	8	1	4	0	3	0	8	0.060	0.001
*bla*OXA-1 (*n* = 14)	8	1	4	0	1	0	6	0.060	0.026
pAmpCs (*n* = 22)	9	3	3	1	5	1	13	0.348	0.000
*bla*DHA-1 (*n* = 10)	7	1	0	0	2	0	3	0.000	0.718
*oqxAB* (*n* = 68)	62	0	2	1	2	1	6	0.000	0.000
*Qnr* (*n* = 62)	37	3	16	1	5	0	25	0.021	0.000
*qnrB4* (*n* = 10)	7	1	0	0	2	0	3	0.339	0.718
*qnrS1* (*n* = 34)	27	1	4	1	1	0	7	0.309	0.996
*aac(6′)Ib-cr* (*n* = 22)	14	0	4	0	3	1	8	1.000	0.038
*rmtB* (*n* = 46)	41	0	2	0	2	1	5	0.001	0.024
*fosA* (*n* = 74)	71	0	0	0	1	2	3	0.000	0.000
*fosA3* (*n* = 36)	31	1	3	0	1	0	5	0.065	0.208

Analysis of ESBL encoding genes found 73 *bla*TEM-1, 7 *bla*CTX-variants, 11 SHV-variants, and 2 *bla*OXA variants. In detail, 7 *bla*CTX-variants were composed of *bla*CTX-M-65 (*n* = 34), *bla*CTX-M-15 (*n* = 18), *bla*CTX-M-14 (*n* = 8), *bla*CTX-M-3 (*n* = 4), *bla*CTX-M-24 (*n* = 2), *bla*CTX-M-55 (*n* = 6) and *bla*CTX-M-45 (*n* = 1). Similarly, 11 SHV-variants were divided into *bla*SHV-11 (*n* = 23), *bla*SHV-12 (*n* = 9), *bla*SHV-28 (*n* = 8), *bla*SHV-182 (*n* = 8), *bla*SHV-13 (*n* = 4), *bla*SHV-1 (*n* = 2), *bla*SHV-67 (*n* = 1), *bla*SHV-36 (*n* = 1), *bla*SHV-172 (*n* = 1), *bla*SHV-15 (*n* = 1) and *bla*SHV-190 (*n* = 1). Two *bla*OXA variants were *bla*OXA-1 (*n* = 14) and *bla*OXA-10 (*n* = 2). Moreover, pAmpCs analysis showed prevalence of *bla*DHA-1 (*n* = 10), followed by 5 *bla*CMY variants, including *bla*CMY-2 (*n* = 4), *bla*CMY-65 (*n* = 2), *bla*CMY-77 (*n* = 2), *bla*CMY-34 (*n* = 1), and *bla*CMY-42 (*n* = 1); *bla*ACT-16 (*n* = 1), *bla*SRT-1 (*n* = 2), *bla*OXY-2-2 (*n* = 1) and *bla*SFO-1 (*n* = 1) were also detected. For PMQRs, there were 68 *oqxAB*, 62 *qnr*-variants, 22 *aac(6′)-Ib-cr* and 1 *qepA*. The variants of *qnr* were as follows: *qnrA1* (*n* = 2), *qnrB4* (*n* = 10), *qnrB7* (*n* = 1), *qnrB18* (*n* = 2), *qnrS1* (*n* = 34), and *qnrS2* (*n* = 3). Finally, 16S-RMTase encoding genes *rmtB* (*n* = 46) and *armA* (*n* = 3), fosfomycin resistant genes *fosA* (*n* = 74), *fosA3* (*n* = 36) and *fosA5* (*n* = 5) were also found.

For the 77 CRKP strains, 70 *bla*KPC-2, 5 *bla*NDM and 6 *bla*CTX-M variants were identified with *bla*CTX-M-65 (*n* = 32), being the predominant resistance determinant, followed by *bla*CTX-M-15 (*n* = 16) and *bla*CTX-M-14 (*n* = 5). Ten *bla*SHV variants containing *bla*SHV-11 (*n* = 18), *bla*SHV-28 (*n* = 8), *bla*SHV-12 (*n* = 7), *bla*SHV-182 (*n* = 7), *bla*SHV-13 (*n* = 4), *bla*SHV-1 (*n* = 1), *bla*SHV-15 (*n* = 1), *bla*SHV-36 (*n* = 1), *bla*SHV-67 *(n* = 1), and *bla*SHV-172 (*n* = 1) were found. The other main genes identified included *bla*TEM-1B (*n* = 60), *bla*DHA-1 (*n* = 7), *bla*OXA-1 (*n* = 8), *rmtB* (*n* = 41), *aac(6′)-Ib-cr* (*n* = 14), *oqxAB* (*n* = 62), *qnrS1* (*n* = 27), and *qnrB4* (*n* = 7). Additionally, 71 (92.2%) out of 77 CRKP strains carried *fosA* genes, among which, 31 (40.2%) co-carried *fosA* and *fosA3*, 5 *fosA5* were also found. Among the 11 CREC*,* there were 3 *bla*KPC-2 and 11 *bla*NDM. Additionally, *bla*KPC-2 was identified in 2 *C. freundii*, 1 *S. marcescens* and 1 *E. cloacae*, *bla*NDM was found in 1 *C. freundii*, 1 *K. oxytoca* and 1 *E. cloacae.*

Overall, *K. pneumoniae* strains carried much more *bla*KPC-2, *bla*CTX-M-65, *bla*TEM-1B, *rmtB*, *fosA*, and *oqxAB* genes compared with other *Enterobacteriaceae*. In particular, *bla*KPC-2, *bla*DHA-1, *fosA*, *oqxAB* and *rmtB* were higher in *K. pneumoniae* than those in *E. coli*. Whereas, more *bla*NDM and *qnr* were distribued among *E. coli* than those in *K. pneumoniae.* As well, more *bla*NDM, *bla*OXA, pAmpCs, *acc(6′)-Ib-cr* and *qnr* in other *Enterobacteriaceae* were prevalent than those in *K. pneumoniae* strains (Table 2)*.* The distribution of resistance determinants among the remaining five no-carbapenemase-producing CRE strains were summarized in Table 3.

**Table 3:** The sequence types and distribution of resistance determinants of CRE strains without producing carbapenemase

code	Strain	STs	CTX-M	SHV	OXA	pAmpC	PQMRs	16S-RMTase	FRG
6	*K. pneumoniae*	ST11	*bla*CTX-M-15	*bla*SHV-28	*bla*OXA-1		*oqxAB*, *qnrS1*, *acc(6′)-Ib-cr*		
54	*K. pneumoniae*	ST15	*bla*CTX-M-15		*bla*OXA-1		*acc(6′)-Ib-cr*		
64	*C. freundii*	ST248	*bla*CTX-M-65, *bla*CTX-M-3	*bla*SHV-11		*bla*CMY-65	*oqxAB*, *acc(6′)-Ib-cr*	*armA*, *rmtB*	
65	*K. oxytoca*	ST105				*bla*CMY-77 *bla*DHA-1	*qnrB18*, *qnrB4*		*fosA3*
933	*K. pneumoniae*	ST290	*bla*CTX-M-15	*oqxAB*		*bla*DHA-1	*qnrB4*		

*PAmpC* Plasmid-mediated AmpC enzyme, *PQMRs* Plasmid-mediated quinolone resistance genes; *16S-RMTase* Exogenously acquired 16S rRNA methyltransferase, *FRG* Fosfomycin resistance gene

### CRE sequence types

Among the 77 CRKP isolates, 13 STs were identified, with ST11 (*n* = 54) being the dominant one, followed by ST15 (*n* = 7) and ST268 (*n* = 5). The other STs included ST942 (*n* = 2), ST48 (*n* = 1), ST290 (*n* = 1), ST1779 (*n* = 1), ST23 (*n* = 1), ST65 (*n* = 1), ST86 (*n* = 1), ST577 (*n* = 1), ST17 (*n* = 1), and ST1 (*n* = 1). There were 7 STs in 8 CREC, which were ST410 (*n* = 2), ST3489 (*n* = 1), ST156 (*n* = 1), ST683 (*n* = 1), ST297 (*n* = 1), ST167 (*n* = 1), and ST361 (*n* = 1). In addition, 3 *E. coli* strains could not be assigned to specific STs. The STs for 4 *C. freundii* strains were ST116 (*n* = 2), ST248 (*n* = 1) and ST36 (*n* = 1). The STs for 2 *K. oxytoca* strains were ST105 and ST36, respectively, and the two *E. cloacae* were assigned to ST418 and ST723.

### Plasmid replicons

The distribution of plasmid replicons was shown in Table 4. Among the 97 CRE strains, 10 strains only carried one plasmid replicon, 82 strains carried two or more plasmid replicons, whereas, 5 strains seemed not to contain any type of plasmids. In total, 15 types of plasmid replicons were detected among the 97 CRE, including IncR (*n* = 64), IncFII (*n* = 63), IncFIB (*n* = 45), IncX3 (*n* = 16), IncFII_K_ (*n* = 13), IncFIA (*n* = 10), IncFIC (n = 2), IncH11 (*n* = 8), IncH12 (n = 4), IncA/C (*n* = 5), IncX1 (*n* = 2), IncN (*n* = 1), IncP1 (*n* = 1), IncB/O (*n* = 1) and IncI1 (*n* = 1). Of these replicon-types, the combination of IncFII and IncR was the most common and mainly found in KPC-2 producers (*n* = 54). Nine types of replicons were detected in CRKP, of which IncR (*n* = 59) dominated, followed by IncFII (*n* = 55), IncFIB (*n* = 34) and IncFII_K_ (*n* = 13). Regarding CREC, 13 types of plasmid replicons were identified, with IncX3 (*n* = 11) and IncFIB (*n* = 8) being the most prevalent types.

**Table 4:** The distribution of major plasmid replicons among carbapenem-resistant *Enterobacteriaceae*

Plasmid replicons	*K. pneumoniae* (*n* = 77)	*K. oxytoca* (*n* = 2)	*E. coli* (*n* = 11)	*S. marcescens* (*n* = 1)	*C. freundii* (*n* = 4)	*E.cloacae* (*n* = 2)	*P* value (*K. pneumoniae* Vs *E. coli*)	*P* value (*K. pneumoniae* Vs *Enterobacter*)
IncR (*n* = 64)	59	2	1	1	0	1	0.000	0.000
IncFII (*n* = 63)	55	0	6	0	1	1	0.256	0.009
IncFIB (*n* = 45)	34	1	8	0	0	2	0.147	0.386
IncX3 (*n* = 16)	2	1	11	0	1	1	0.000	0.000
IncFII_K_ (*n* = 13)	13	0	0	0	0	0	0.052	0.011
IncFIA (*n* = 10)	7	0	3	0	0	0	0.204	0.718

Compared with other *Enterobacteriaceae* species, *K. pneumoniae* had obviously higher distribution of IncFII, IncR and IncFII_K_ (*p* < 0.05). In contrast, the distribution of IncX3 was lower in the *K. pneumoniae* strains than those among other *Enterobacteriaceae* species (*p* < 0.05).

No significant differences were shown on the distributions between some main resistance determinants and specific plasmid replicons among the CRE (Table 5), such as *bla*NDM, *bla*CTX-M-15, *bla*DHA-1, *qnrB4* and *aac(6′)-Ib-cr* with IncX3, IncFIIk, and IncFIA, *bla*CTX-M and *oqxAB* with IncFII and IncR, *bla*CTX-M-65 with IncFIB, pAmpCs with IncX3, *rmtB* and IncFIB, *fosA* with IncR, as well as *fosA3* with IncFIB (Table 5). Furthermore, the consistency on most of these distributions could also be observed among the CRKP (Table 6), and more detailed distribution correlations were further displayed between the following resistance determinants and plasmid replicons: *bla*CTX-M-15 with IncFII_K_, *qnr* and *qnrS1* with IncFIB, *rmtB* with IncFIB, *fosA3* with IncFIB, *aac(6′)-Ib-cr* with IncFII_K_.

**Table 5:** The differences on the distribution of plasmid replicons and resistant determinants among the carbapenem-resistant *Enterobacteriaceae*

Resistance determinants	Plasmid replicons					
	IncFII (*n* = 63)	IncR (*n* = 64)	IncFIB (*n* = 45)	IncX3 (*n* = 16)	IncFII_K_ (*n* = 13)	IncFIA (*n* = 10)
*bla*KPC-2 (*n* = 77)	0.001	0.005	0.000	0.000	0.000	0.000
*bla*NDM (*n* = 19)	0.000	0.000	0.000	0.754	1.000	0.503
*bla*CTX-M (*n* = 74)	0.229	0.440	0.002	0.000	0.000	0.000
*bla*CTX-M-15 (*n* = 18)	0.000	0.000	0.000	0.701	0.210	0.093
*bla*CTX-M-65 (*n* = 34)	0.000	0.000	0.124	0.026	0.002	0.001
*bla*SHV (*n* = 59)	0.665	0.568	0.093	0.000	0.000	0.000
*bla*SHV-11 (*n* = 23)	0.000	0.000	0.002	0.487	0.087	0.019
*bla*TEM (*n* = 73)	0.087	0.121	0.000	0.000	0.000	0.000
*bla*TEM-1B (*n* = 71)	0.185	0.281	0.000	0.000	0.000	0.000
*bla*OXA (*n* = 16)	0.139	0.000	0.000	0.824	0.000	0.839
*bla*OXA-1 (*n* = 14)	0.000	0.000	0.000	0.481	1.000	1.000
pAmpCs (*n* = 22)	0.000	0.000	0.000	0.122	0.043	0.008
*bla*DHA-1 (*n* = 10)	0.000	0.000	0.000	0.503	1.000	0.815
*oqxAB* (*n* = 68)	0.720	1.000	0.009	0.000	0.000	0.000
*qnr* (*n* = 62)	0.235	0.275	0.262	0.000	0.000	0.000
*qnrB4* (*n* = 10)	0.000	0.000	0.000	0.701	1.000	0.648
*qnrS1* (*n* = 34)	0.000	0.000	0.073	0.009	0.000	0.000
*aac(6′)Ib-cr* (*n* = 22)	0.000	0.000	0.000	0.557	0.230	0.087
*rmtB* (*n* = 46)	0.004	0.014	0.659	0.000	0.000	0.000
*fosA* (*n* = 74)	0.017	0.152	0.000	0.000	0.000	0.000
*fosA3* (*n* = 36)	0.000	0.000	0.322	0.002	0.001	0.000

**Table 6:** The differences on the distribution of plasmid replicons and resistant determinants among the carbapenem resistant *K. pneumoniae*

Resistance determinants	Plasmid replicons				
	FII (*n* = 55)	R (*n* = 59)	FIB (*n* = 34)	FII_K_ (*n* = 13)	FIA (*n* = 7)
*bla*KPC-2 (*n* = 70)	0.001	0.007	0.000	0.000	0.000
*bla*CTX-M (*n* = 59)	0.572	1.000	0.000	0.000	0.000
*bla*CTX-M-15 (*n* = 16)	0.000	0.000	0.001	0.629	0.049
*bla*CTX-M-65 (*n* = 32)	0.000	0.000	0.890	0.005	0.000
*bla*SHV (*n* = 49)	0.405	1.000	0.000	0.000	0.000
*bla*SHV-11 (*n* = 18)	0.510	0.000	0.011	0.424	0.027
*bla*TEM (*n* = 60)	0.405	1.000	0.000	0.000	0.000
*oqxAB* (*n* = 62)	0.839	0.845	0.001	0.000	0.000
*Qnr* (*n* = 37)	0.025	0.002	0.690	0.000	0.000
*qnrS1* (*n* = 27)	0.000	0.000	0.210	0.009	0.000
*aac(6′)Ib-cr* (*n* = 14)	0.000	0.000	0.167	1.000	0.000
*rmtB* (*n* = 41)	0.003	0.000	0.324	0.000	0.000
*fosA* (*n* = 71)	0.000	0.004	0.000	0.000	0.000
*fosA3* (*n* = 31)	0.000	0.000	0.742	0.008	0.000

## Discussion

In this study, we provided data on the antimicrobial resistance profiles, STs, and plasmid replicons profiles of CRE isolates collected from specialized hospitals, Children’s hospital and level II hospitals in Nanjing district, Southeast China.

Most of our CRE strains displayed high resistance against the commonly used antimicrobial agents, which was consistent with previous report, providing evidence that the CRE strains are usually resistant to many other classes of antibiotics in clinical practice [24]. Fortunately, most of the CRE strains were still susceptible towards tigecycline, colistin, ceftazidime/avibactam, and aztreonam/avibactam. Resistance to aztreonam/avibactam and ceftazidime/avibactam have appeared in our study, which may be attributed to mutations in the carbepenamase KPC and NDM [25], suggesting a rapid resistance development of *K. pneumoniae*. None carbepenemase encoding genes were detected among the 5 CRE strains in our study, where the production of ESBLs variants and/or AmpC enzymes in combination of overexpressed efflux pumps or the decreased permeability might contribute to the carbapenem resistance.

The high prevalence of *bla*KPC-2 and many other resistance related genes, such as *bla*ESBLs, PMQRs, as well as 16S-RMTase was consistent with our previous report [8], indicating the frequent co-existence or co-evolution of antibiotic resistance genes, which might lead to the emergence of untreatable *K. pneumoniae* infections. Among them, the quite high prevalence of *bla*ESBL among the *bla*KPC-2 producing CRE strains in our study represented crippling and urgent threats to public health, because multiple copies of *bla*CTX-M and *bla*KPC within plasmids could be integrated and disseminated into chromosome [26]. In that case, the spread of such strains would be horizontally and vertically accelerated within hospitals. To date, the co-occurrence of *bla*CTX-M-65 and *bla*KPC-2 in our study has been previously reported in *K. pneumoniae* [27]. Notably, we found quite high prevalence of *fosA* in *bla*KPC-2 producing *K. pneumoniae. fosA* has been reported to be chromosomally encoded by clinically relevant Gram-negative species and contributes to intrinsic fosfomycin resistance [28]. However, some strains carrying *fosA* in our study displayed susceptibility to fosfomycin, which needs to be further investigated. Furthermore, the wide distribution of *fosA* and *fosA3,* as well as the emergence of *fosA5,* indicated that fosfomycin should be cautiously used for treating the CRKP infections, since the combination of plasmid-borne *fosA3* and *bla*KPC-2 could accelerate the spread of antibiotic resistance [29]. Another interesting point is that, *bla*CTX-M-45 used to be identified by an algorithm [30]. However, this is the first time that we found this gene in a clinical *C. freundii* isolate. Among these SHV encoding genes, the *bla*SHV-13 was discovered in *K. pneumoniae* isolates in Amsterdam [31], whereas, the *bla*SHV-67, *bla*SHV-172, *bla*SHV-182, and *bla*SHV-190 are brand new, and have never been reported previously. OXA-10-type class D β-lactamases (previously shown to be weak carbapenemases) was firstly identified in our clinical *C. freundii* [32]. Moreover, CMY-34 was identified in *C. freundii* isolate in Danish army recruits, and was also found in our study [33]. To the best of our knowledge, the two *bla*CMY-65 producing *C. freundii* strains, one *bla*CMY-77 producing *K. pneumoniae* isolate, one *bla*CMY-77 producing *K. oxytoca* and a *bla*ACT-16 producing *E. cloacae* found in our study have not been reported previously. For the first time, we found the co-occurrence of *bla*DHA-1 and *bla*CMY-65 in *C. freundii*, and the co-occurrence of *bla*CMY-77 and *bla*DHA-1 in *K. oxytoca.*

The expansion of ST11 for the KPC-2 producing *K. pneumoniae* was in accordance with previous report indicating that the ST11 is the dominating epidemic clone among CRKP [15]. Albeit the KPC-2 producing *E. coli* ST410 has been reported, as far as we know, the *bla*NDM-5 and *bla*KPC-2 co-carrying *E. coli* ST167, *bla*NDM-5 carrying *K. oxytoca* ST36, as well as *bla*IMP-4 and *bla*NDM-1 co-carrying *E. cloacae* ST418 identified in our study have not been reported previously. Additionally, the *bla*KPC-2 has been identified in *C. freundii* and *E. cloacae*, and *bla*NDM-1 in *C. freundii*. This is the first time that we identified *bla*KPC-2 in *C. freundii* ST116 and *E. cloacae* ST723, and *bla*NDM-1 in *C. freundii* ST36.

Plasmids are extra-chromosomal DNA elements representing major reservoirs for horizontal transmission of antibiotic resistance among bacteria [34]. To date, multiple plasmids have been found to be the vesicles for spread of carbapenemase, ESBLs, and PMQRs [34]. The high prevalence of IncFII, IncR and IncFIB plasmid replicons in our study alert us on the urgency of implementing antimicrobial resistance surveillance, since IncFII-type plasmids are highly distributed vesicles for resistant determinants in *Enterobacteriaceae* [35]; Moreover, IncR plasmid is an important reservoir of multidrug resistance in *Enterobacteriaceae* strains, because the conserved IncR backbones include the multidrug resistant (MDR) regions [36]; and IncFIB plasmids were reported to be associated with majority of the antimicrobial resistance genes [37]. Similarly, some of the distribution correlations between various plasmid replicons and multiple resistant genes found in our study are consistent with the previous reports [38–40]. Among them, IncX3 plasmid is a narrow-spectrum plasmid widely distributed in *E. coli* from wildlife in Europe [38], which has also been found to be predominantly associated with fluoroquinolone resistance genes and β-lactam resistance genes. This is the similar situation in our CRE strains, where IncX3 was also found to be associated with *bla*NDM and *qnrB4*. However, the situation in *K. pneumoniae* is still uncertain, because few IncX3 plasmid replicons were found. In addition, plasmids encoding carbapenemases have been demonstrated to play a core role in the rapid spread of CRE [39]. In our study, the distribution correlations between *bla*NDM and plasmid replicons IncX3, IncFIA and IncFII_K_ also confirmed the proposal, since the spread of *bla*NDM is involved in diverse and heterogeneous plasmids [40]. As we know, IncX3 plasmid has been an important vehicle with high mobility in worldwide dissemination of *bla*NDM [41], and IncFIA-type conjugative plasmid encoding *bla*NDM-1 has been involved in the outbreak caused by *K. pneumoniae* strains in Tunisia [11]. The IncN and IncH1 plasmids in our study are less common, this is in accordance with the previous study, which revealed that IncN and IncH1 plasmids are host-specific, and are more predominant in livestock and horses in Denmark, respectively [42]. However, the appearance of such plasmid replicons in our study may suggest the spread of plasmids among human and farm livestock.

Five isolates lacked any plasmid type in our study, which may be due to the limitations in the PBRT protocol [43]. It’s however noteworthy that the co-existence of multiple plasmid replicons in our CRE strains is consistent with previous study [39], which may result from a conserved backbone responsible for regulation and mating pair stabilization [44].

## Conclusion

In addition to wide distribution of CRE isolates in Nanjing district, Southeast China, the high-level carriage of carbapenemases, ESBLs, pAmpCs, 16S-RMTase, PMQRs and major plasmid replicons in our study might be a potential challenge regarding the transmissible capability. Moreover, the expansion of ST11 *bla*KPC-2 carrying *K. pneumoniae*, the new emergence of *bla*NDM-5 carrying *K. oxytoca* ST36, as well as *bla*IMP-4 and *bla*NDM-1 co-carrying *E. cloacae* ST418 are worrying, efficient and sustained control measures are urgently required.

## Data Availability

The datasets used and/or analyzed during the current study are available from the corresponding author on reasonable request.

## Abbreviations

16S-RMTases: 16S rRNA methyltransferase
CRE: Carbapenem-resistant *Enterobacteriaceae*
CREC: Carbapenem resistant *Escherichia coli*
CRKP: Carbapenem resistance *Klebsiella pneumoniae*
ESBLs: Extended-spectrum β-lactamases
KPC: *Klebsiella pneumoniae* carbapenemase
MIC: Minimum inhibitory concentration
MLST: Multi-locus sequence typing
NDM: New Delhi metallo-ß-lactamase
PBRT: PCR-based replicon typing
PCR: Polymerase Chain Reaction
PMQRs: Plasmid mediated quinolone resistance genes
STs: Sequence types
